# Three Decades of Silicosis: Disease Trends at Autopsy in South African Gold Miners

**DOI:** 10.1289/ehp.0900918

**Published:** 2009-11-23

**Authors:** Gill Nelson, Brendan Girdler-Brown, Ntombizodwa Ndlovu, Jill Murray

**Affiliations:** 1 National Institute for Occupational Health, National Health Laboratory Service, Johannesburg, South Africa; 2 School of Public Health, University of the Witwatersrand, Johannesburg, South Africa; 3 School of Health Systems and Public Health, University of Pretoria, South Africa

**Keywords:** Africa, mining, PATHAUT, pneumoconiosis, silica

## Abstract

**Background:**

Eliminating silicosis is a priority of the International Labour Organization and the World Health Organization. Prevalence is particularly high in developing countries.

**Objectives:**

We describe trends in silicosis among South African gold miners who had had an autopsy between 1975 and 2007 and quantify the contributions of age at autopsy and employment duration to these trends.

**Methods:**

South African miners and ex-miners are eligible for autopsy examination for occupational lung disease, regardless of the clinical cause of death, and the families of deceased mine workers may receive compensation from the government of South Africa. Miners who died from external causes and who had been employed in the gold mines for > 1 year were stratified by population group because of differences in exposure, patterns of employment, and autopsy referral patterns. We extracted data from PATHAUT (Pathology Automation System) and used Stata 10 to estimate trends in relative proportions of silicosis that were standardized for age and employment duration.

**Results:**

The crude proportion of silicosis for white miners was six times that of black miners in 1975. By 2007, it was 1.5 times higher for black miners. The proportion of miners with silicosis increased from 0.03 to 0.32 for black miners and from 0.18 to 0.22 for white miners. The increase can be explained by increasing age and employment duration for white miners. For black miners, it can be only partly explained by these two factors.

**Conclusion:**

As miners continue to age and work for longer periods, the burden of silicosis will continue to rise. South Africa is committed to global efforts to eliminate silicosis by 2030. The autopsy database allows for disease surveillance, which is necessary to monitor the success of this initiative.

Silicosis is a major occupational health concern in both developed and developing countries. Although disease rates are reported to be decreasing in some developed countries (Bang et al. 2008; Gerhardsson 2002), silicosis is still very common in low- and middle-income countries (Lehtinen and Goldstein 2002), and mining countries have particularly high prevalences of silicosis (Rees and Murray 2007). South Africa joined the International Labour Organization and the World Health Organization (WHO) Global Program for the Elimination of Silicosis and has developed a national initiative under the leadership of the Department of Labour. In 2003, the Mine Health and Safety Council developed its own milestone, namely, that after December 2013, using present diagnostic techniques, no new cases of silicosis will occur among previously unexposed individuals (individuals unexposed before 2008) (Mine Health and Safety Council 2007).

South African legislation provides mineworkers who were ever employed in the mines of South Africa the right to have their cardiorespiratory organs examined and their families to be compensated for occupational lung disease, regardless of the cause of death (Occupational Diseases in Mines and Works Act of 1973). Although the autopsy service is used by approximately 80% of all miners who die while employed and by many white ex-miners, very few black ex-miners have had an autopsy. Autopsies are performed at the National Institute for Occupational Health (NIOH) in Johannesburg, and data on pathologic diagnoses, personal information (demographics), and work histories, including the commodities mined and the length of time spent in each, have been stored in an electronic database known as PATHAUT (Pathology Automation System; National Institute for Occupational Health, Johannesburg, South Africa) since 1975 (Hessel et al. 1987). The database currently contains more than 100,000 records and provides an ideal information source to analyze trends in respiratory disease over the past three decades. Of the miners who have come to autopsy, approximately 80% have worked in the gold mines, with employment ranging from 1 month to several decades.

During the past three decades, the gold mining industry has employed hundreds of thousands of workers, most of whom were black underground miners. Employment peaked in the mid-1980s at around 550,000. In the 1970s and 1980s, most black miners were migrant workers from rural areas of South Africa and from neighboring countries, employed on relatively short contract periods of around 18 months. When their contracts expired, they returned to their homes, after which they could apply for a new contract, although many men found employment elsewhere. On the other hand, white miners were employed as career miners. During the last two decades, this pattern has slowly changed as the contract system fell away and black miners became increasingly employed as career miners, although many are still migrants.

Historically, black miners have been exposed to higher dust levels than were white miners. Black men are employed underground in high-dust occupations such as drilling and stoping, whereas white men are largely employed in supervisory positions and maintenance jobs with lower dust exposure.

The only study on the prevalence of silicosis among white gold miners in South Africa is a large cohort of more than 2,200 men who were employed for an average of 24 years from 1940 to the early 1970s. By 1991, the prevalence of radiologically diagnosed silicosis was 14% (Hnizdo and Sluis-Cremer 1993). Murray and Hnizdo (unpublished data) analyzed the cohort autopsy data for an exposure–response relationship and found silicosis in almost 52% of the deceased mine workers who had been exposed to dust for 40 years.

Several cross-sectional studies have been conducted on black gold miners since the early 1990s, either on employed miners (Churchyard et al. 2004) or on ex-miners (Girdler-Brown et al. 2008; Steen et al. 1997; Trapido et al. 1998). Only one study has analyzed time trends in silicosis, in black gold miners who died of external causes while in mine employment (Murray et al. 1996).

In the present study we examined long-term trends in the proportions of both black and white South African gold miners with silicosis, who died from external causes. We used autopsy data from miners and ex-miners, which we standardized for age at death and duration of employment in the analysis.

## Materials and Methods

### Study population

Gold miners were defined as those who had ever been employed in the gold mining industry. The study population comprised all gold miners who had been employed for > 1 year and who died from an external cause of death. External causes of death included those coded in the *International Classification of Diseases* (WHO 1992) as injuries (e.g., as a result of mine or traffic accidents), burns, poisoning, drowning, intentional self-harm, and homicide. The following variables were extracted from the PATHAUT database for 1975–2007: population group, duration of gold mining employment, age at death, and whether silicosis was present at autopsy.

Miners have been significantly affected by the human immunodeficiency virus (HIV) epidemic during the past two decades, and tuberculosis is one of the most common causes of death in this HIV-affected population group (Murray et al. 2007). Miners with tuberculosis are more likely to have silicosis than those without (Corbett et al. 2000). Furthermore, the presence of silicosis increases the risk of mortality from tuberculosis, which is exacerbated by HIV infection (Churchyard et al. 2000). Thus, we felt that including those who died from causes other than external causes might bias the results of the trend analysis, and they were excluded from the study population.

### Diagnosis of silicosis

Silicosis was defined as the presence of palpable silicotic nodules on macroscopic examination of the lungs, which was then confirmed on microscopic examination. The diagnoses were made by experienced pathologists.

### Statistical analyses

Gold miners were stratified by population group (black and white) because of their differing employment, dust exposure, and autopsy referral patterns. Potential risk factors for the development of silicosis were considered to be age at death and duration of employment in the gold mining industry (as proxy measures of dust exposure in the absence of dust measurements), and year of autopsy.

All data analyses were carried out using Stata 10, (version 10; StataCorp LP, College Station, TX, USA) except 95% confidence intervals (CIs) for standardized proportion ratios (SPRs), which were calculated manually. Trends in the crude proportions of miners with silicosis by year were assessed by means of simple linear regression with weighting by the inverse of the variances of the single year proportions. Binary logistic regression modeling with silicosis (1 = present, 0 = absent) as a dichotomous outcome was used to estimate associations with the explanatory variables.

We compared the proportions of black and white miners with silicosis by 5-year intervals using direct standardization for age and duration of employment. Age categories that were selected for the standardization were < 40, 40–49, 50–59, and ≥ 60 years. Duration of employment categories were < 10, 10–14, 15–19, 20–24, 25–29, and ≥ 30 years. Age-group– and duration-group–specific proportions were then weighted by the total number of miners (black and white combined) in each age and duration category in the calculation of the standardized proportions with silicosis by year of autopsy interval. The variances for the year of autopsy interval black and white standardized proportions were calculated using the binomial approximation method without corrections for finite populations (Cochran 1977). The exact 95% CIs for the SPRs were calculated using the formula given by Curado et al. (2007).

We performed the same direct age and duration of employment standardized analysis of the proportions of miners with silicosis for those who died from nonexternal causes. The standardization was carried out using the same weightings that were used for those dying from external causes to enable comparisons to be made between the two groups.

The modeling process was carried out with the three explanatory variables (year of autopsy, age at death, and duration of employment) coded as categorical variables. Modeling was attempted with all possible combinations of coding options for these three variables (continuous vs. categorical), but none of the models that included one or more of the variables as a continuous variable had resulting acceptable goodness-of-fit test results. Interaction terms for year and age, year and duration, and age and duration were also included in the initial models for black and white miners.

Initially, age group categories were made up of 10-year intervals and duration group categories were made up of 5-year intervals. However, some categories were subsequently collapsed because of very small numbers of cases in some of the groups. In addition, we combined the first three age group categories for white miners because we found no statistically significant difference between them.

Postregression analysis for the binary logistic regression model included the Pearson’s goodness-of-fit test and, as appropriate (if the number of covariate pattern cells with expected values < 5 exceeded 10% of all the cells), the Hosmer-Lemeshow goodness-of-fit test, as well as calculation of the area under the receiver operating characteristic (ROC) curve (Hosmer and Lemeshow 2000). Decisions to drop any explanatory variable from the binary logistic regression models were based on a significant Likelihood Ratio test with an alpha value of 0.05.

The study was approved by the University of the Witwatersrand Human Ethics Committee (protocol M050228).

## Results

Of the 98,323 miners in the PATHAUT database, 76,231 (77.5%) had ever worked in the gold mining industry. Of these, 50,867 (66.7%) were black and 25,282 (33.2%) were white. The remaining 82 miners were classified as belonging to another population group, or information about their population group was missing or could not be validated.

A total of 19,143 miners died from external causes and worked for > 1 year in the gold mining industry. Table 1 lists the numbers of subjects excluded from the analysis and the reasons for their exclusion. The final study population comprised 16,411 black miners and 2,732 white miners.

The mean age at death of the black miners was 35.0 years (median, 34 years; range, 17–82 years), whereas the mean age at death of the white miners was 48.3 years (median, 47 years; range, 18–96 years). For the black miners, the mean age at death rose from 33.0 years in 1975 to 43.4 years in 2007, whereas the mean age at death for the white miners rose from 44.1 years in 1975 to 54.4 years during the same period.

Table 2 lists the age distributions of the study subjects, by population group, together with the age-specific proportions of silicosis found at autopsy. The proportion of black miners with silicosis had reached 0.18 by 40–49 years, more than double that of white miners in the same age group. The proportion of miners ≥ 60 years old with silicosis was higher among white miners than among black miners.

The average duration of employment in gold mining for the black miners was 7.7 years (median, 6.1 years; range, 1.1–48.0 years), and that for white miners was 17.1 years (median, 15.0 years; range, 1.1–54.0 years) for white miners. During the study period, the mean duration of employment increased from 5.6 to 13.4 years for the black miners, whereas that for the white miners increased from 17.5 to 20.1 years.

Table 3 illustrates the distribution of study subjects by duration of employment, by population group, together with the relevant proportions of miners with silicosis for each duration group. As the duration of employment increased among black and white miners, so did the proportion with silicosis. The proportion of black miners with silicosis reached 0.22 after 15–19 years of employment, but reached only 0.20 after 20–24 years in white miners. There was silicosis in both black and white miners who had been employed for < 10 years.

The results presented in Tables 2 and 3 show the crude proportions of miners with silicosis. The crude proportion, for the entire study period, was lower for black miners (0.08) than for white miners (0.14). However, after direct standardization for age and duration of employment, and applying the population group-specific proportions by age and duration of employment to this combined sample, the standardized proportions with silicosis changed to 0.09 for black miners and 0.05 for white miners (Table 4). This represents a change in the ratio of these proportions from 0.56 for the crude data to 1.70 for the proportions standardized for age and duration of employment. We calculated these ratios using estimates that were not rounded off; thus, they differ slightly from those based on the rounded-off estimates in the tables.

The differences between the population group standardized proportions for miners who died from external causes (Table 4) were not statistically significant for the initial period 1975–1979 or for 2000–2007. However, for the remaining 5-year time intervals, the proportions of black miners with silicosis were significantly higher than those of white miners, with SPRs of 2–3. The two periods for which the differences were not statistically significant are somewhat unusual, in that the crude proportion for black miners in the first period was lower than that for the other periods, whereas that for white miners in the last period was higher than for the other periods. It is apparent, however, from these results, that population group behaves as an effect modifier of the relationship between calendar interval and proportion with silicosis for the period 1980–1999.

Table 5 shows the age and duration of employment-standardized proportions of miners with silicosis for those miners who died from nonexternal causes. The differences between the population group standardized proportions were statistically significant for all periods other than 1975–1979. The proportions of black miners with silicosis were significantly higher than those for white miners for the remaining 5-year intervals.

In addition, although the year group-specific standardized proportions with silicosis were very similar for the white miners who died from nonexternal causes, we found an increasing trend in these proportions for black miners during the period of the study.

The standardized proportion of white miners with silicosis was very similar for those who died from external and nonexternal causes (0.06 and 0.05, respectively, with a ratio of 1.11; 95% CI, 0.95–1.29). However, for the black miners with silicosis, the proportion of those who died from nonexternal causes (0.13; 95% CI, 0.09–0.10) was much higher than for those who died from external causes (0.09; 95% CI, 0.12–0.13). The ratio of these two standardized proportions was statistically significant (1.48; 95% CI, 1.37–1.60). We calculated these ratios using nonrounded estimates; thus, they differ slightly from those calculated from the rounded estimates.

**Figure 1:**
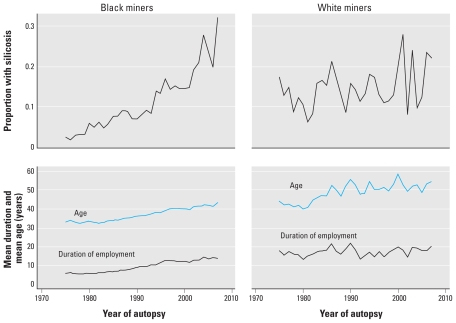
Crude population group-specific proportions of gold miners with silicosis (external causes of death): mean ages and mean employment durations from 1975 to 2007 (*n* = 16,411 for black miners and *n* = 2,732 for white miners).

The population-group–specific trends in the proportions of miners with silicosis who died from external causes are illustrated in Figure 1. These are crude proportions for the period 1975–2007. The proportion of white miners with silicosis was six times higher than that of black miners in 1975. By 2007, the proportion was 1.5 times higher for black miners. The proportion of black gold miners with silicosis increased steadily from 0.03 in 1975 to 0.32 in 2007 (trend assessment using the variance weighted least squares method: *p* < 0.001). The proportion of white gold miners with silicosis increased from 0.18 to 0.22 (*p* = 0.16) during the same period.

We first modeled the data (using logistic regression modeling) as a single data set with the inclusion of a dichotomous variable for population group. The resulting model proved to have poor goodness-of-fit statistics (*p* < 0.01). We then stratified the data by population group and developed two separate logistic regression models.

Tables 6 and 7 present the results of the binary logistic regression analysis. None of the interaction terms was statistically significant in the models for the black or the white miners. Consequently, we dropped the interaction terms from both models. The results show increasing adjusted odds ratios (ORs) for disease (relative to the baseline groups) as age groups increase for both the black and the white miners. In addition, the adjusted ORs for disease increase (relative to the baseline groups) for both population groups as the durations of exposure increase, apart from white miners with more than 34 years of exposure, where the OR falls to 6.71.

For black miners, all year-of-autopsy groups have raised adjusted ORs, relative to the period 1975–1979, of 1.90–2.94. For the white miners, however, all the year-of-autopsy category–adjusted ORs were not statistically significant (data not shown). This result indicates a lack of evidence that the year-to-year risks have changed for these miners during the study period after adjusting for age and duration of employment. Hence, we omitted the variable for year of autopsy from the model in Table 7.

Silicosis is classified by severity in the PATHUAT database. We analyzed these data but found no trends with regard to severity during the study period.

## Discussion

Our results show that the overall proportion of gold miners with silicosis at autopsy is currently high at 0.32 for black miners and 0.22 for white miners (2007 data). In the late 1990s, two groups reported similarly high prevalences in two cross-sectional studies of living black ex-miners, ranging from 22% to 36%, depending on the X-ray reader (Trapido et al. 1998; Steen et al. 1997). More recently, Churchyard et al. reported prevalences of 18.3% to 19.9% in employed black miners older than 37 years (Churchyard et al. 2004). Girdler-Brown et al. found a prevalence of 24.6% in black ex-miners with a mean employment duration of 26 years (Girdler-Brown et al. 2008). The high prevalence of 51.6% in white miners, calculated by Murray and Hnizdo, was for a cohort of deceased miners employed for an average of 40 years (Murray and Hnizdo, unpublished data).

The frequency distributions of the miners in the different age categories and duration of employment categories were very different in the two population groups. This may be why the attempt at modeling the data as a single data set with an added dichotomous variable for population group was unsuccessful. Having two separate regression models for the two population groups, each with somewhat different age groups and duration of employment groups, means that the effect of population group on the proportion with silicosis is difficult to discern from the regression results.

We investigated this issue by performing age group and duration group direct standardization of the proportions of black and white miners with silicosis. After standardizing for these factors, the proportion of black miners with silicosis was almost double (1.7-fold) that of white miners for the overall study period and for those who died from external causes. The difference was even greater for those who died from nonexternal causes (2.27-fold).

These findings lend support to our belief that the inclusion of data from the miners who died from nonexternal causes would have biased our findings. The HIV epidemic has resulted in an increase in tuberculosis and an associated increase in mortality; those with tuberculosis are more likely to have silicosis than those without (Corbett et al. 2000).

The higher proportions of black miners with silicosis, after adjusting for age and duration of employment, suggest that black miners had higher intensities of exposure to silica than white miners. Generally, black miners are employed as drillers and stopers, and these are the processes that generate the most dust. White men, on the other hand, are exposed to less dust in their jobs as supervisors and maintenance workers. This is supported by the fact that the proportion of black miners with silicosis reached 0.2 after shorter durations of employment than for white miners. In addition, a much higher proportion of black miners had silicosis at younger ages than did white miners. The proportion of black miners younger than 50 years with silicosis was more than double that of white miners in the same age group.

In addition to the high proportion of miners with silicosis, this study shows that the proportions in black miners have been rising since the mid-1970s. The proportion of black miners with silicosis increased more than 10-fold during the study period. This increase can be partly attributed to increasing age at autopsy and longer employment periods, both of which may be direct consequences of workforce stabilization, a socioeconomic phenomenon that started in the 1980s. In the 1970s, when the computerized autopsy database was established, the system of employing miners on short contracts was in force. Gradually, economic changes led to increased poverty in the rural areas and fewer opportunities for alternative employment. Stabilization of the workforce increased (Cowie and van Schalkwyk 1987; Leger 1992), and more black miners became career miners. Before the mid-1980s, black miners with tuberculosis were discharged and repatriated to the rural areas and neighboring countries after being started on treatment, to reduce the number of ill miners in the workforce. When short-course chemotherapy for tuberculosis was introduced into the gold mines, they were allowed to continue working (Cowie 1989), and this also resulted in longer employment periods. In this study, the mean age at autopsy among black miners increased by > 10 years, from 33 to 43.4 years, whereas mean duration of employment increased by almost 8 years, from 5.6 to 13.4 years.

Year of autopsy is the year in which the pathologic examination was performed and correlates with the year of death. Because year of autopsy remained a significant predictor of silicosis among black miners when we adjusted the data for duration of employment and age at death, it is likely that there are other factors that we cannot measure that contribute toward this increase. As technology advanced over the years, and higher speed rock drills were developed, it is possible that the dust particles that were generated became smaller. It is also possible that, as mine shafts became deeper, the surface properties and hence the toxicity of the dust increased, or there may have been an increase in the proportion of freshly fractured silica dust. The gradual introduction of production bonuses may also have resulted in men working for longer shifts, or for more shifts each year, which would not be reflected in the duration variable that measures exposure in calendar months.

Limiting the study population to those who died from external causes did not change the results with regard to the trends during the study period. When we included all causes of death, the crude proportions of black miners increased from 0.03 in 1975 to 0.34 in 2007. In those dying of external causes, these proportions were very similar (0.03 and 0.32, respectively).

Some miners in both population groups developed silicosis within 10 years of employment. This is likely to be accelerated silicosis, which usually occurs within 10 years of dust exposure. It is not acute silicosis (silica- associated alveolar proteinosis); we have seen only a single case in the last 25 years, and this was in a nonminer.

The increase in ORs was more strongly associated with age than with duration of employment. It is well established that silicosis progresses even after cessation of dust exposure (Hessel et al. 1988). The high OR in older white men, in particular, may be due to longer residence time of dust in the lungs.

The strengths of the present study include that it is population-based and that the study population comprises around 19,000 gold miners for whom data were collected for a period of more than 30 years. In addition, the diagnosis of silicosis was made at autopsy, rather than radiologically, by experienced pathologists, using standardized methods. Autopsies are useful for diagnosing diseases such as silicosis that may be undetected on X rays. Corbett et al. (1999) found radiology to be insensitive for detecting early silicosis, using miniature X rays. Hnizdo et al. (1993), using standard sized films, also showed lower radiologic sensitivity compared with autopsy findings, even for advanced silicosis.

There are several potential biases in the data. One is that there is a difference between black and white miners with regard to the use of the autopsy service. Most white men who die while employed or after having left the mining industry come to autopsy. The proportion of white gold miners coming to autopsy is high, at more than 80%. In 1986, Hessel et al. (1986) estimated that 86% of deceased white gold miners undergo autopsy. This is supported by a cohort study in which around 1,700 gold miners who had started working in the 1940s were followed up for several decades (Murray and Hnizdo, unpublished data). By 2003, 83% of the deceased men had had an autopsy.

The rate is also high for black miners who died while employed. Corbett et al. (1999) reported that 94% of gold miners, employed by a single company, who died in the period January 1996 to June 1997 came to autopsy. In a more recent study on causes of death in a large cohort of black gold miners, “70% of the deaths occurring in the mine were followed by autopsy” (Murray et al. 2007). Very few men in this study who died elsewhere came to autopsy (7%).

Often, black miners who retire or leave the mining industry for other reasons return home to the rural areas of South Africa and neighboring countries from where they were recruited, often far from medical services that are able to remove the cardiorespiratory organs for autopsy. Miners who develop disabling silicosis while employed are not allowed to continue to work in dusty jobs, and so they leave the mines. Thus, the missing black miners may have had more disease (as a proportion) as a result of heavier or longer exposures (healthy worker effect). Because elderly, retired black miners seldom come to autopsy, the black miners are generally younger at autopsy than are the white miners. In this study, few autopsies had been conducted on black miners who were older than 59 years or who had worked for > 24 years.

These biases will underestimate the proportion of black miners with silicosis. The NIOH has an outreach program that addresses miners’ rights regarding autopsy examination and ascertaining compensation for occupational lung disease. This program has been extended to the rural areas of South Africa and neighboring countries.

The main limitation in this study was the unavailability of dust measurements and the use of proxy measurements of age at autopsy and duration of employment to estimate cumulative dust exposures.

There is no evidence that silica dust levels have decreased in the mines over the past few decades. In 1994, the Commission of Inquiry into Safety and Health in the Mining Industry, led by Judge R.N. Leon, concluded that “dust levels have remained roughly the same over a period of about 50 years” (South African OHS Commissions 1995). There is no reason to believe that they have decreased since the commission’s inquiry (Churchyard et al. 2004).

The increasing proportion of black gold miners with silicosis at autopsy has several implications. Prevalences of diseases associated with silicosis and silica dust exposure, such as tuberculosis, chronic obstructive airway disease (COAD), and lung cancer, are also likely to rise. No data exists on trends in lung cancer or in COAD for South African gold miners, but the burden of COAD in gold miners is high (Girdler-Brown et al. 2008). The most important impact, however, is the prevalence of tuberculosis among black miners, which is increasing (Sonnenberg et al. 2000). The HIV epidemic has played a major role in the rising prevalence of tuberculosis but has been exacerbated by the presence of silicosis, because silicosis and HIV have a multiplicative effect on tuberculosis (Corbett et al. 2000).

## Conclusion

Our analyses show that, during the 33-year study period, there has been no reduction in the proportion of miners (with external causes of death) coming to autopsy with pathologic evidence of silicosis after adjustment for age group and duration of employment. Furthermore, the standardized proportion of black miners with silicosis is almost double that of white miners during this period. The large proportion of men with silicosis reflects the inability of gold mining companies to reduce silica dust to safe levels. As miners age and work for longer periods, the burden of silicosis and its associated diseases will continue to rise. HIV, tuberculosis, and silicosis have a multiplicative interaction. Thus, the high proportion of South African gold miners with silicosis, many of whom are already burdened with tuberculosis and/or HIV, has far reaching implications in terms of health services that need to be prepared for increasing morbidity and mortality rates in both current and ex-miners. South Africa is committed to global efforts to eliminate silicosis by 2030, but this will require extraordinary efforts. The recording of valid and reliable dust measurements linked to ongoing medical surveillance is essential for success.

## Figures and Tables

**Table 1:** Numbers of gold miners on PATHAUT database and resons for exclusions.

Exclusion	No.	Remaining
Ever worked in gold mining industry		76,231
No age at death or birth date	2,727	73,504
No duration of gold mining employment	11,145	62,359
Race other than black or white	65	62,294
Validation of data not possible	21	62,273
Age at first employment < 15 years	194	62,079
Duration of employment ≤ 1 year	4,818	57,261
Cause of death not “external”	38,118	19,143^a^

a Includes 16,411 black miners and 2,732 white miners.

**Table 2:** Crude proportions with silicosis at autopsy of miners, by age at death, stratified by population group: 1975–2007 (external causes of death).

	Black			White		
Age group (years)	*n*	Silicosis present	Proportion	*n*	Silicosis present	Proportion
< 30	5,285	27	0.00	386	1	0.00
30–39	6,234	328	0.05	529	17	0.03
40–49	3,561	635	0.18	587	45	0.08
50–59	1,184	283	0.24	535	95	0.18
60–69	133	28	0.21	374	120	0.32
> 69	14	3	0.21	321	113	0.35
Total	16,411	1,304	0.08	2,732	391	0.14

**Table 3:** Crude proportions with silicosis at autopsy of miners, by duration of employment, stratified by population group: 1975–2007 (external causes of death).

Duration of employment (years)	Black			White		
	*n*	Silicosis present	Proportion	*n*	Silicosis present	Proportion
< 10	11,683	443	0.04	978	19	0.02
10–14	2,815	377	0.13	362	28	0.08
15–19	1,284	286	0.22	320	40	0.13
20–24	400	119	0.30	306	60	0.20
25–29	161	61	0.38	265	74	0.28
> 30	68	18	0.26	501	170	0.34
Total	16,411	1,304	0.08	2,732	391	0.14

**Table 4:** Age- and duration-standardized proportions of miners with silicosis, by population group and time interval (external causes of death), with time interval-specific SPRs (blacks to whites).

	Black			White			
Year of autopsy	*n*	Proportion with silicosis		*n*	Proportion with silicosis		SPR^a^ (95% CI)
		Crude	Standardized (95% CI)		Crude	Standardized (95% CI)	
1975–1979	3,167	0.03	0.04 (0.04–0.05)	487	0.13	0.05 (0.03–0.07)	0.90 (0.63–1.20)
1980–1984	3,795	0.06	0.08 (0.07–0.09)	487	0.11	0.04 (0.03–0.05)	2.19 (1.60–2.99)
1985–1989	4,205	0.08	0.11 (0.10–0.12)	669	0.16	0.04 (0.03–0.05)	2.69 (1.94–3.72)
1990–1994	2,968	0.09	0.09 (0.08–0.09)	549	0.15	0.04 (0.03–0.06)	1.98 (1.34–2.94)
1995–1999	1,321	0.14	0.10 (0.09–0.12)	258	0.13	0.03 (0.02–0.05)	2.98 (1.99–4.47)
2000–2007	955	0.20	0.12 (0.10–0.14)	282	0.18	0.13 (0.06–0.20)	0.96 (0.56–1.63)
All years	16,411	0.08	0.09 (0.09–0.10)	2,732	0.14	0.05 (0.04–0.06)	1.70 (1.45–1.99)

a SPRs indicate ratios of the standardized proportion of black miners with silicosis to the standardized proportion of white miners with silicosis.

**Table 5:** Age- and duration-standardized proportions of miners with silicosis, by population group and time interval (nonexternal causes of death), with time interval-specific SPRs (blacks to whites).

	Black			White			
Year of autopsy	*n*	Proportion with silicosis		*n*	Proportion with silicosis		SPR^a^ (95% CI)
		Crude	Standardized (95% CI)		Crude	Standardized (95% CI)	
1975–1979	2,054	0.07	0.06 (0.05–0.07)	3,629	0.31	0.06 (0.05–0.07)	0.95 (0.72–1.27)
1980–1984	1,884	0.13	0.11 (0.09–0.12)	3,890	0.32	0.07 (0.04–0.09)	1.66 (1.18–2.34)
1985–1989	2,010	0.16	0.12 (0.10–0.13)	3,512	0.26	0.05 (0.03–0.07)	2.44 (1.55–3.82)
1990–1994	2,670	0.18	0.12 (0.10–0.13)	3,118	0.24	0.05 (0.03–0.06)	2.57 (1.70–3.89)
1995–1999	3,192	0.23	0.14 (0.12–0.15)	2,284	0.22	0.05 (0.04–0.06)	2.71 (2.01–3.67)
2000–2007	6,466	0.29	0.16 (0.15–0.17)	3,409	0.24	0.06 (0.04–0.09)	2.56 (1.66–3.95)
All years	18,276	0.21	0.13 (0.12–0.13)	19,842	0.27	0.06 (0.05–0.06)	2.27 (2.12–2.43)

a SPRs indicate ratios of the standardized proportion of black miners with silicosis to the standardized proportion of white miners with silicosis.

**Table 6:** Adjusted ORs for black miners (external causes of death).

Variable	*n*	OR	SE	*z*-Value	*p*-Value	95% CI
Age group (years)						
< 30	6,113	Reference				
30–39	5,921	7.40	1.26	11.74	< 0.001	5.30–10.34
40–49	3,315	20.25	3.45	17.65	< 0.001	14.50–28.29
≥ 50	1,062	22.08	4.03	16.94	< 0.001	15.43–31.58

Duration of employment (years)						
< 10	11,683	Reference				
10–14	2,815	1.93	0.15	8.37	< 0.001	1.65–2.25
15–19	1,284	2.54	0.23	10.18	< 0.001	2.12–3.03
≥ 20	629	3.06	0.34	10.18	< 0.001	2.47–3.80

Year of autopsy						
1975–1979	3,167	Reference				
1980–1984	3,795	2.02	0.27	5.35	< 0.001	1.56–2.61
1985–1989	4,205	2.43	0.30	7.12	< 0.001	1.90–3.10
1990–1994	2,968	1.90	0.25	4.98	< 0.001	1.48–2.45
1995–1999	1,321	2.27	0.32	5.88	< 0.001	1.73–2.99
2000–2007	955	2.94	0.42	7.60	< 0.001	2.22–3.88

Area under the ROC curve = 0.82. Hosmer-Lemeshow goodness-of-fit test *p*-value = 0.36 (10 groups), 0.13 (8 groups), and 0.35 (12 groups). Pearson’s goodness-of-fit test *p*-value = 0.01; when performing the Pearson goodness-of-fit test, 25 of 168 (14.9%) of the expected cell values were < 5, so the Hosmer-Lemeshow test result is preferred.

**Table 7:** Adjusted ORs for white miners (external causes of death).

Variable	*n*	OR	SE	*z*-Value	*p*-Value	95% CI
Age group (years)						
< 50	1,555	Reference				
50–59	519	2.62	0.48	5.27	< 0.001	1.83–3.75
60–69	378	5.41	1.00	9.13	< 0.001	3.76–7.77
≥ 70	280	6.07	1.21	9.04	< 0.001	4.11–8.97

Duration of employment (years)						
< 10	978	Reference				
10–14	362	3.62	1.12	4.17	< 0.001	1.98–6.62
15–19	320	5.30	1.55	5.69	< 0.001	2.98–9.42
20–24	306	8.13	2.28	7.47	< 0.001	4.69–14.08
25–29	265	10.37	2.91	8.35	< 0.001	5.99–17.97
30–34	260	11.98	3.37	8.84	< 0.001	6.91–20.78
≥ 35	241	6.71	1.95	6.55	< 0.001	3.79–11.85

Year of autopsy groups is not shown because all ORs were not statistically significant. Area under the ROC curve = 0.82. Pearson goodness-of-fit test *p*-value = 0.38; Hosmer-Lemeshow goodness-of-fit test *p*-value = 0.79 (10 groups), 0.62 (8 groups), and 0.46 (12 groups); when performing the Pearson goodness-of-fit test, 5 of 54 (9.3%) of the expected cell values were < 5.
